# Controllable Melting and Flow of Ag in Self-Formed Amorphous Carbonaceous Shell for Nanointerconnection

**DOI:** 10.3390/mi13020213

**Published:** 2022-01-29

**Authors:** Zhiqiang Yu, Qing Shi, Huaping Wang, Junyi Shang, Qiang Huang, Toshio Fukuda

**Affiliations:** 1 Beijing Advanced Innovation Center for Intelligent Robots and Systems, School of Mechatronical Engineering, Beijing Institute of Technology, Beijing 100081, China; shiqing@bit.edu.cn (Q.S.); wanghuaping@bit.edu.cn (H.W.); qhuang@bit.edu.cn (Q.H.); tofukuda@nifty.com (T.F.); 2 Key Laboratory of Biomimetic Robots and Systems, Beijing Institute of Technology, Ministry of Education, Beijing 100081, China; 3 School of Automation, Beijing Institute of Technology, Beijing 100081, China; shangjunyi@bit.edu.cn

**Keywords:** nanorobotic system, core–shell nanostructure, metal-filled nanotubes, mass transport, electromigration force

## Abstract

Nanointerconnection has been selected as a promising method in the post-Moore era to realize device miniaturization and integration. Even with many advances, the existing nanojoining methods still need further developments to meet the three-dimensional nanostructure construction requirements of the next-generation devices. Here, we proposed an efficient silver (Ag)-filled nanotube fabrication method and realized the controllable melting and ultrafine flow of the encapsulated silver at a subfemtogram (0.83 fg/s) level, which presents broad application prospects in the interconnection of materials in the nanometer or even subnanometer. We coated Ag nanowire with polyvinylpyrrolidone (PVP) to obtain core–shell nanostructures instead of the conventional well-established nanotube filling or direct synthesis technique, thus overcoming obstacles such as low filling rate, discontinuous metalcore, and limited filling length. Electromigration and thermal gradient force were figured out as the dominant forces for the controllable flow of molten silver. The conductive amorphous carbonaceous shell formed by pyrolyzing the insulative PVP layer was also verified by energy dispersive spectroscopy (EDS), which enabled the continued outflow of the internal Ag. Finally, a reconfigurable nanointerconnection experiment was implemented, which opens the way for interconnection error correction in the fabrication of nanoelectronic devices.

## 1. Introduction

Functional nanomaterials have been widely used in a variety of applications such as sensing [1,2], energy harvesting [3,4,5], and medical treatments [6] due to their unique physicochemical performance compared with conventional bulk counterparts. Current challenges in this field are the accurate assembly and rapid integration of these functional nanomaterials for various application requirements. In the perspective of practical application, accuracy and reliable electrical interconnection between functional nanomaterials and circuits (electrodes) play a crucial role in the final performance of the assembled devices. The electrical interconnection of functional nanomaterials frequently implies the construction of a conductive nanojunction. The conventional choices to accomplish this assignment are scanning electron microscope (SEM)-based methods (electron beam-induced deposition (EBID) [7] and Joule heating fusion [8]) and transmission electron microscopy (TEM) or atomic force microscope (AFM)-based approaches (cold welding [9,10] and spot welding [11]).

Metallic precursors rich in gold (Au), silver (Ag), platinum (Pt), tungsten (W), etc., elements have been widely used in the EBID systems, which are sufficient for multiple applications in low-throughput nanotechnology. However, using metallic precursors suffers from some limitations, including the toxicity of the precursors, the presence of residues left by the precursors, the proximity effects, and the relatively low deposition rates [7]. In addition, the interconnection junctions made by the EBID are not reconfigurable [12]: once the junction is fabricated, it is practically impossible to remove. Unlike the metallic precursor’s deposition methods, the Joule heating fusion method is not only useful in the fabrication of high-throughput in-plane transparent film/electrodes but also promising in the spatial nanostructure interconnection with the assistance of the nanorobotic system [8].

Compared to the interconnection techniques inside the SEM, the TEM system provides enhanced magnification of the samples and enables various refined interconnection operations of functional nanomaterials. Benefitting from this setup, a cold welding technique [10] was proposed and observed inside the TEM due to the diffusion of the metallic atoms, which are known for their spontaneous formation of nanojunctions. However, this diffusion process puts forward high requirements for the cleanliness level of the functional nanomaterial surface and can only work in metallic nanomaterials. In general, a larger surface-area-to-volume ratio allows the functional nanomaterials to more easily absorb contaminants or dispersants on their surface, such as PVP, which in turn hampers the use of this technique.

Spot welding [11] predicts a promising interconnection technique in nanoscale and can be understood by tracking back to the developed welding methods in the macroscale. It always requires a robotic system to carry a “gun” to provide the interconnection material and a local field to achieve their transmission. Metal-filled carbon nanotubes have been developed as an attempt to act as the so-called gun while an electric field (electromigration force) was adopted as the local field to realize the transport of the metal inside the nanotubes. With the help of a nanorobotics system, spot welding demonstrates a high dimensional and spatially accurate interconnection ability and can overcome the obstacles that exist in the conventional nanointerconnection methods. However, the complex metal filling process and the low filling rate of the metal limit the development of spot welding. In addition, the extremely limited TEM chamber hampers the integration of the multi-degree-of-freedoms (DOFs) nanorobotic system, which ultimately hinders the broadening of this method.

Our work introduces a new rapid Ag filling method for spot welding based on a 16 DOFs nanorobotic system inside an SEM and realizes the controllable melting and flow of encapsulated Ag. Compared to TEM, the larger SEM chamber enables the manipulation and interconnection of more complex nanostructures. To our knowledge, we are the first to realize nanoscale spot welding inside an SEM chamber by using a metal-filled nanotube. Thanks to the size effect, we successfully obtained the amorphous carbon (a-C)-coated Ag nanowire (NW) before the evaporation of its encapsulated Ag by pyrolyzing the PVP-coated Ag NWs. The controllable melting and flow of the Ag inside the a-C shell are also investigated by adjusting the current density in the wrapped Ag NW. The product of the PVP pyrolysis is also characterized by the TEM-EDS, which validates the formation of the a-C shell. Based on the controllable melting and flow properties, we implemented a reconfigurable interconnection experiment which showed the ability to bridge nanowires with a size down to 50 nm, and the potential for subnanometer structural interconnection. Ultimately, we briefly discuss the possible applications of spot welding that, in the near future, will broaden the catalogue of the existing techniques for nanofabrication.

## 2. Materials and Methods

### 2.1. Materials

The Ag NW was purchased from XFNANO (XFJ02, Nanjing, China; solvent: IPA, 20 mg/mL) with an average diameter of 50 nm. The PVP powders (K26-35, MREDA, Beijing, China) were used as received. A 1 wt.% IPA solution of PVP was mixed with the Ag NW by a mixer at 2000 rpm for 1 min. Thanks to the large surface-area-to-volume ratio, the PVP can easily adhere to the Ag NW surface via the strong coordinative field of nitrogen and oxygen [13]. As the coordinate bond formation between PVP and silver ions, a PVP protective layer is generated spontaneously. The multiwalled carbon nanotube (MWCNT) powder was purchased from Suzhou Carbon Feng Graphene Technology Co., Ltd. (HQNANO-CNTs-013, Suzhou, China). Pure tungsten nanowire (150 µm in diameter) was purchased from TKS (W31-B, Suzhou, China). Sodium hydroxide was purchased from Meryer and used without further purification. The SourceMeter (2635B, Keithley, Tektronix Inc., Beaverton, OR, USA) was employed to pyrolyze the PVP and investigate the controllable melting and flow of the encapsulated Ag. The TEM (JEM-2100, JEOL Ltd., Akishima, Japan) was employed to obtain the morphology and constituent elements of the pyrolysis products of PVP.

### 2.2. Nanorobotic System

A 16 DOFs nanomanipulation system was integrated into the specimen stage of an ESEM (Quanta 650 FEG, FEI, Hillsboro, OR, USA). The nanorobotics system has four quadrants of four degrees-of-freedom nanomanipulators, each consisting of 3 linear positioners (SLC-1720-s, SmarAct, Oldenburg, Germany) to move along the *X*, *Y*, and *Z* axes with a 12 mm stroke and a rotation positioner (SR-2013, SmarAct, Oldenburg, Germany) to obtain an endless rotation of the end effectors for observation and operation. The resolution of the linear and rotation positioner is 1 nm and 2 µ°, respectively. Additionally, the intrinsic specimen stage of the ESEM consists of a 5-dimensional coarse stage along the *X*, *Y* axes within 150 mm, the *Z* axis within 65 mm, the tilting (T) axis within −5° to +70°, and the rotation (R) axis within 360°. It is mainly used to position the samples and the end effectors in the field of view (FOV). By introducing the electric signal through a feedthrough into the SEM chamber, an electrical in situ characterization nanorobotic system was constructed.

### 2.3. Tungsten Probe Fabrication

Several homemade tungsten probes (probe diameter: ~150 µm; tip diameter: ~150 nm) were used in this study to pick-place and characterize the nanowires. They were chemically etched using a 2 mol/L NaOH solution on a homemade automatic etching machine. The initially given etching length was 3 mm, and a 3.5 v bias was applied on the tungsten wire while elevating the tungsten wire at a rate of 1 mm/min. After 3 min, the probes were immersed in the ethyl alcohol solution and sonicated for 1 min before being mounted on the probe holder for any nanomanipulation.

### 2.4. Assembly the MWCNT–Ag NW Nanostructure

The basic steps required for the construction of the MWCNT–Ag NW nanostructure can be described as: (i) Preparing a tailored copper grid for detailed morphology characterization of the MWCNT–Ag NW nanostructure in TEM; (ii) Picking up an individual MWCNT and transferring it into the custom copper grid for suspending the Ag NW; (iii) Picking up a single PVP-coated Ag NW and carefully attaching it to the transferred MWCNT to form a physical contact.

## 3. Results

**Figure 1:**
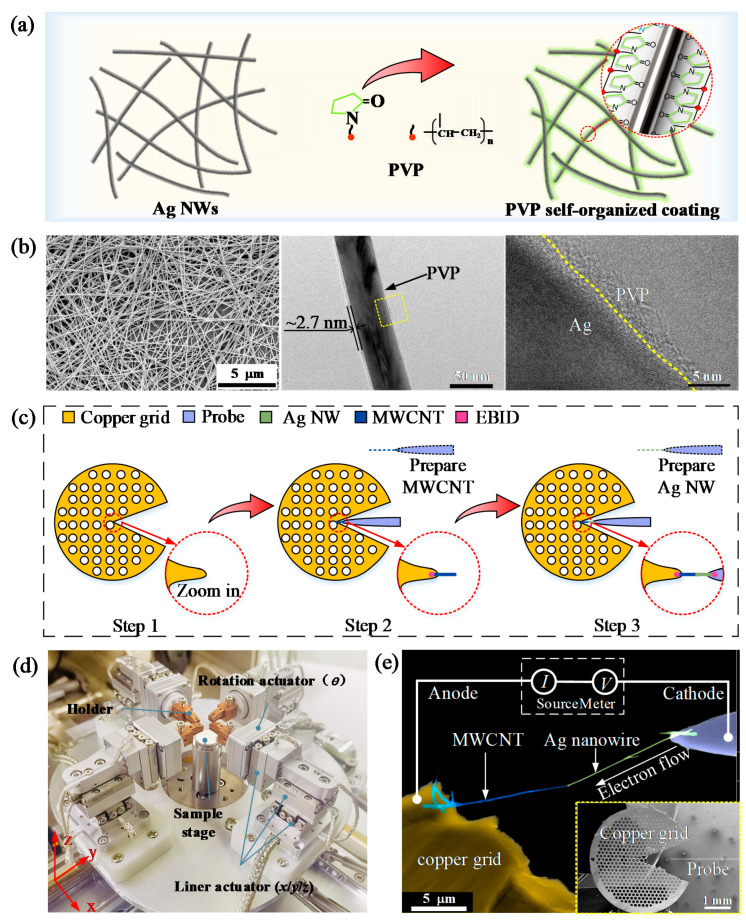
Characterization of PVP-coated Ag NWs and assembly of the MWCNT–Ag NW nanostructure for nanointerconnection. (**a**) Schematic illustration of the self-organized coating of PVP on Ag NWs; (**b**) SEM image (**left**), TEM image (**middle**), and high-resolution TEM (HRTEM) (**right**) image of the PVP-coated Ag NWs; (**c**) Schematic illustration of the MWCNT–Ag NW nanostructure assembly; (**d**) Nanorobotics system; (**e**) Final constructed MWCNT–Ag NW nanostructure. The copper grid is regarded as the anode while the tungsten probe is the cathode.

To comprehensively understand the process of PVP pyrolysis, and subsequently the melting and flow of encapsulated Ag, we first assembled the MWCNT–Ag NW nanostructure and investigated the melting and flow process of the encapsulated Ag under SEM through a 16 DOFs nanorobotics system [14], and then obtained its morphology and the constituent elements of the products through high-resolution TEM (HRTEM) image and EDS mapping results in TEM. Figure 1a describes the self-organized PVP coating process of Ag NWs, while Figure 1b (left) demonstrates the typical SEM image of the PVP-coated Ag NWs. The morphology of a single PVP-coated Ag NW was also characterized under TEM in our previous study [8] (Figure 1b, middle), indicating that the thickness of the PVP layer was ~2.7 nm. We also characterized the interaction surface between the PVP layer and its encapsulated Ag NW through HRTEM [8]. We can see that the PVP is strongly adsorbed on the surface of the Ag NW (Figure 1b, right).

The stable mechanical contact was verified by shifting the Ag NW a distance (e.g., ~100 nm) perpendicular to its axis. If the MWCNT moves together with the Ag nanowire, we learn that mechanical contact is formed. In general, the Ag NW has three typical contact cases with the MWCNT: end-to-side, end-to-end, and side-to-side. We can see that they all need to pyrolyze a ~2.7 nm PVP layer no matter which contact case. In our research, we adopt the end-to-side mode (end: Ag NW; side: MWCNT). The final assembled MWCNT–Ag NW nanostructure is demonstrated in Figure 1e. Here, the copper grid is regarded as the anode while the tungsten probe is the cathode. The MWCNT is used as a support to suspend the Ag nanowire for further morphology details characterization in TEM. When the MWCNT and Ag NW were brought into mechanical contact by the nanorobotics system (Figure 1d), a sequence of sweep voltage was applied on this nanostructure for pyrolyzing the PVP and melting the encapsulated Ag. As the applied sweep bias voltage increased, the sweep time first decreased and then stabilized, indicating a negligible effect on the final applied constant bias voltage.

### 3.1. Controllable Melting of the Encapsulated Ag

**Figure 2:**
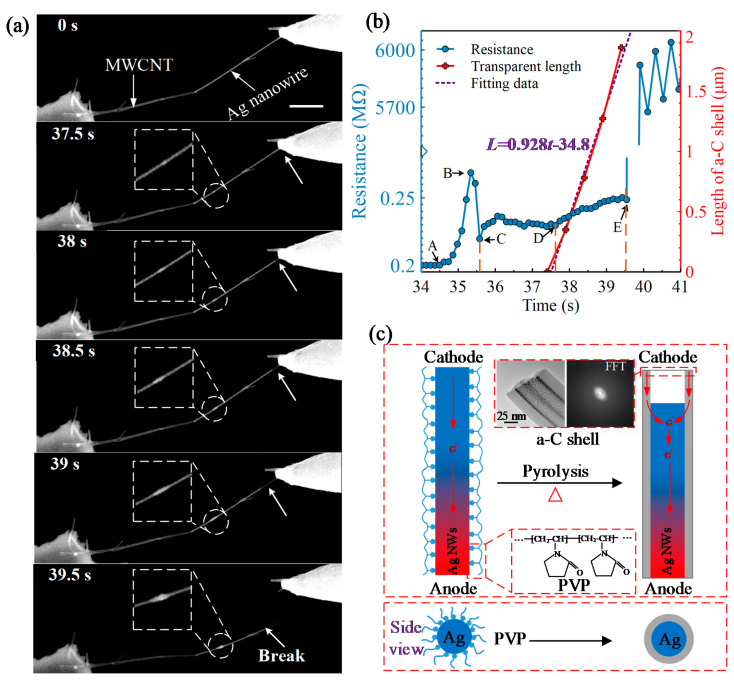
Controllable melting of the encapsulated Ag inside PVP shell. (**a**) Time-resolved melting and flow of the wrapped Ag. Scale bar: 2 µm; (**b**) Melting and flow state detection of the internal Ag based on resistance variation. The AC stage indicates the phase transition of the encapsulated Ag while the CD and DE stages represent the melting and flow states of Ag, respectively; (**c**) Schematic illustration of transparent shell formation and Ag flow mechanism. The inset HRTEM image (**left**) predicts the enlarging porous a-C shell, and its corresponding fast Fourier transformation (FFT) image is shown on the right.

To figure out the proper loading bias, we first loaded an increasing sweep voltage on the MWCNT–Ag NW nanostructure from 0 V to 14 V with 1 V steps. When the total resistance of this nanostructure drops to hundreds of kiloohms, we consider the maximum loaded sweep voltage as the proper voltage. As such, we obtained an appropriate voltage of 14 V. After that, a constant DC bias of 14 V was loaded on the MWCNT–Ag NW nanostructure to replace the sweep voltage. Meanwhile, the morphology of the MWCNT–Ag nanostructure was recorded simultaneously in real-time through the video. Figure 2a depicts the time-resolved morphology changes of the assembled nanostructure when we loaded the constant voltage. Notably, the tungsten probe here is brighter than the copper grid, which indicates the tungsten probe is connected to the cathode of the power. An Ag junction occurred and grew gradually with time, which indicates the melting of the encapsulated Ag. More interestingly, a transparent shell was formed and can further provide the current for the melting and flow of Ag. As is well known, the intrinsic PVP is an insulator and cannot supply the current transmission. Therefore, we believe that a conductive shell has been formed before the flow of Ag.

Figure 2b demonstrates the time-resolved resistance curve and the transparent nanotube lengthening velocity. The formation of the transparent nanotube is due to the outflow of the inside Ag, which reflects the flow velocity of the internal Ag. By carefully observing the time-resolved curve of the resistance, we note that the whole process can be divided into three stages. For the first stage (AB and BC), we find the total resistance undergoes a rapid increase in the AB stage before the flow of the Ag (no transparent nanotube generated). This change is similar to that found in Ref. [15]. We attribute this abrupt increase in resistance to the phase change of Ag. In most cases, these local transient configurations with larger resistance are not stable, and the resistance tends to drop to a stable value, which explains the decrease in resistance in the BC stage. For the second stage (CD), the resistance is maintained at ~230 KΩ, which is greater than the stable resistance before point A (~200 KΩ). According to the different resistivities of solid Ag (1.59 × 10^−8^ Ωm) and melting Ag (1.8 × 10^−7^ Ωm) [16], we can easily explain the increase of resistance in stage CD. For the last stage (DE), the molten Ag begins to flow and induces the gradual increase of the total resistance by leaving the transparent shell, which inherently possesses a higher electrical resistivity. Moreover, point D, at which the resistance starts to raise, is almost the same as the time the molten Ag starts to flow. The linear increase of resistance and the transparent shell with time indicates that the increased resistance at the DE stage is due to the lengthening of the transparent shell. The total length of the formed transparent shell in Figure 2b is ~1.86 µm. Considering the mean diameter of the transparent shell and encapsulated Ag NW is around 75.45 nm and 54.54 nm, respectively, the resistivity of the transparent shell can be calculated as ~2.28 × 10^−5^ Ωm, using the resistance difference (20 KΩ) between 37.5 s and 39.5 s. This resistivity of the transparent shell is on the same order as a-C (~1.6 × 10^−5^ Ωm). We verified the formation of the a-C shell in terms of the carbon source, crystallinity, and constituent element weights. From Figure 1a, we have learned that the PVP molecule is mainly composed of C, which can be treated as a carbon source due to its high carbon content. The crystallinity and the weight of constituent elements will be further verified by HRTEM images and EDS mapping results in the following section. After point E, the nanostructure breaks due to the excessive Joule heat. The broken nanostructure can be found in Figure 2a (at 39.5 s). We also calculate the flow velocity of the Ag by fitting the time-resolved transparent shell curve as 928 nm/s. In the meanwhile, the average mass flow rate calculated according to the density of the molten Ag (9.32 g/cm^3^) is 20.20 fg/s. Based on the observed average current (52.47 × 10^−6^ A) and the average of the total resistance (2.29 × 10^5^ Ω), we also calculated the generated Joule heat to be 6.30 × 10^−4^ J.

For the sake of clarity, we explain the formation of the transparent shell and Ag flow mechanism, as shown in Figure 2c. The inset HRTEM image in Figure 2c and its FFT image verify the amorphous state of the newly formed transparent shell. With the Joule heat injection, the temperature increases gradually and finally triggers the pyrolysis of the PVP layer. During this process, the loss of PVP oxygen-containing groups usually generates micropores and stays inside of the transparent a-C shell, which can produce a thicker a-C layer compared to coated PVP layer [17]. As such, a ~2.7 nm PVP layer generated an enlarged ~10 nm thickness a-C shell.

As for the flow mechanism of Ag, multiple mechanisms have been reported, such as the electromigration force [18,19,20,21,22], the irradiation of the electron beam [23], the thermal gradient force [24], and the capillary force [25,26,27]. In general, the irradiation of the electrons from the electron beam can induce the temperature rise of the Ag NW due to the inelastic scattering. However, no melting or morphological changes of the Ag NW or PVP layer have been observed before loading the bias voltage. The thermal gradient force results from the difference of the temperature and in the direction from high temperature to low temperature. In our experiment, the Joule heating is mainly located on the MWCNT due to its larger resistivity (on the order of 10^−6^ Ωm) at the initial stage. Thus, the thermal gradient force is from the MWCNT to Ag, which is contrary to the observed flow of the Ag. As the generation of the a-C shell next to the probe, a new thermal gradient force occurred due to the formation of the transparent shell, facilitating the flow of Ag from the probe to MWCNT. This is because the resistivity of Ag is on the order of 10^−8^ Ωm, which is much lower than that of the a-C nanotube (on the order of 10^−5^ Ωm). Therefore, the a-C shell will be bypassed until the internal Ag flows through. In other words, due to the good heat dissipation conditions of the probe, an opposite thermal gradient force occurs at the initial state between the PVP-coated Ag NW and the probe when compared to the observed results. Capillary force can indeed induce the flow of the molten Ag. However, it is insufficient to break the newly formed a-C nanotube and produce a growing junction.

The electromigration force generated by the high-density directional electron flow agrees well with our experiments, as shown in Figure 2c. The current during the flow of Ag is observed in our experiment at approximately 50 μA. Hence, the current density can be obtained according to the area of the cross-section of Ag NW as 2.14 × 10^6^ A/cm^2^. This result is much lower than those reported in Ref. [28] (2.0 × 10^8^ A/cm^2^). There is no doubt that the thermal gradient plays a considerable effect on the flow of the Ag. Beyond this, the Joule heat from the MWCNT and the a-C shell also increase the activity of the Ag atoms. Taken together, the electromigration force is assumed to be dominant for the directional flow of the internal Ag while the thermal gradient force facilitates the process.

### 3.2. Controllable Flow of the Melting Ag and Product Characterization

**Figure 3:**
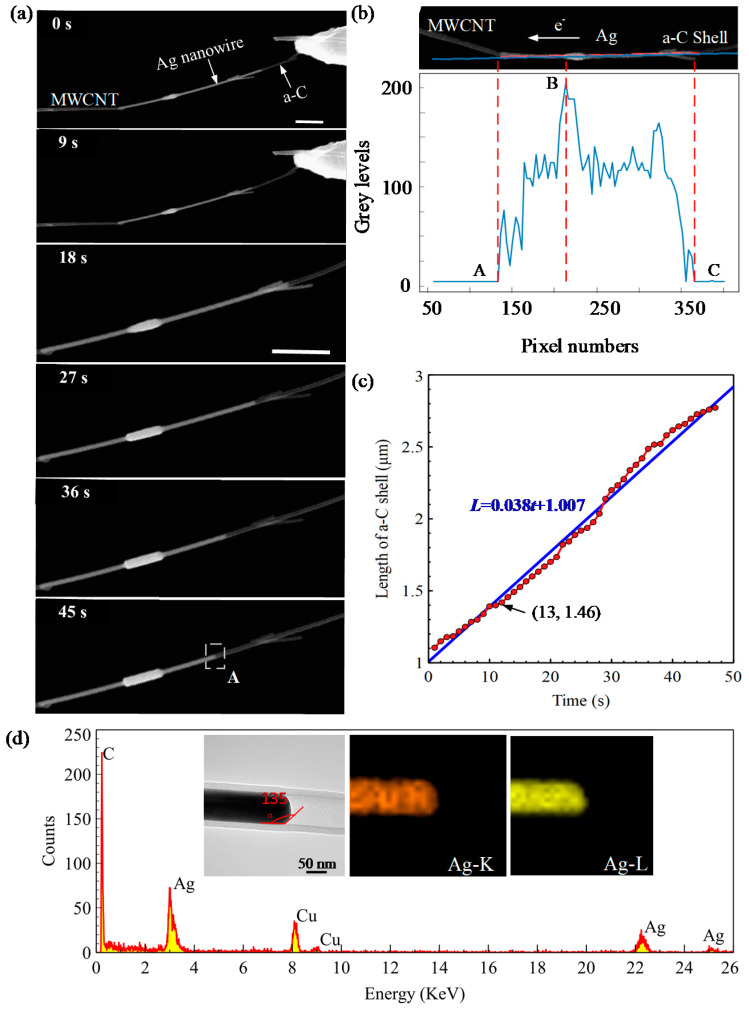
The controllable flow of the encapsulated Ag and elemental characterization of the transparent shell from PVP pyrolysis. (**a**) Time-resolved flow of the wrapped Ag. Scale bar: 1 µm; (**b**) Transfer velocity extraction of the molten Ag based on image processing; (**c**) Time-resolved mass transport velocity of the molten Ag; (**d**) Morphology and constituent elements study of the newly formed core–shell nanostructure. The inset images demonstrate the typical TEM image of the a-C shell and its constituent elements. The EDS mapping area (400 nm × 400 nm) is highlighted in (**a**) at 45 s by a white dotted line.

We reconnected the broken a-C shell and reduced the limit current to 30 µA while keeping the same loading bias. Figure 3a depicts a series of time-resolved SEM images of the Ag flow process at different times. We see that the earlier formed junction continues to grow and finally form a uniform columnar junction due to the directional flow of the internal Ag. To accurately calculate the flow velocity of the Ag, the transparent length of the a-C nanotubes over time was calculated by image processing based on the recorded video frames.

Figure 3b is the video frame highlighted by the point (13, 1.46) in Figure 3c. The variation of the pixel grey value along the Ag-filled a-C nanotube is presented in Figure 3b bottom from a linear fitting method. The total length (5538.0 nm) of the Ag-filled a-C nanotube can be measured from Figure 2a. The transient shell remaining length (226 × 18.01 = 4070.26 nm, where 18.01 is the pixel size) was calculated based on the difference of pixel numbers (226 pixels) from point A (137 pixels) to point C (363 pixels). Therefore, the transient length of the a-C shell (5538.0−4070.26 = 1467.74 nm) can be obtained by subtracting the remaining length of the Ag from the total length of the Ag-filled a-C nanotube. Figure 3c demonstrates the flow velocity of the molten Ag. The flow velocity of the Ag can be determined by fitting the data of the transparent length of the a-C shell, yielding approximately 38 nm/s. More interestingly, this velocity is not affected by the growing resistance of the newly formed a-C shell. Compared to the flow velocity of Ag under 50 µA, the flow velocity is greatly reduced (approximately 1/24 times). In this case, the average mass flow rate of Ag can be calculated as 0.83 fg/s. The current density here can also be achieved according to the area of the cross-section of Ag NW as 1.28 × 10^6^ A/cm^2^. Notably, the current density is effective for controlling the mass flow rate of Ag. It is foreseeable that this subfemtogram mass flow rate is slow enough for precise application, such as spot welding or the nanoprinting of metal in additive manufacturing nanotechnology.

To comprehensively understand the properties of the a-C shell, EDS mapping was used to analyze the compositional elements of the a-C shell. The EDS mapping area was highlighted by a white dashed rectangle in Figure 3a at 45 s. The TEM image of this range is also demonstrated in the inset image (left) of Figure 3d. We note that all the Ag can outflow the a-C shell and form a contact angle between the a-C shell and Ag at 135°. We attribute the large contact angle to the weak bonding energy between the carbon and Ag atom. This hydrophobic property provides a good interface condition for the encapsulated Ag to completely flow out the a-C shell. From the EDS mapping results, we observe that two main elements were gathered (i.e., C and Ag), which indicates the pyrolysis product of the PVP is rich in the C element. The Ag element is derived from encapsulated Ag (inset EDS mapping image, Ag-K, Ag-L). Considering the spatial interaction volume (on the order of 10^−6^ m) of EDS [29], the extra element Cu was observed due to the use of the copper grid.

### 3.3. Reconfigurable Nanointerconnection

**Figure 4:**
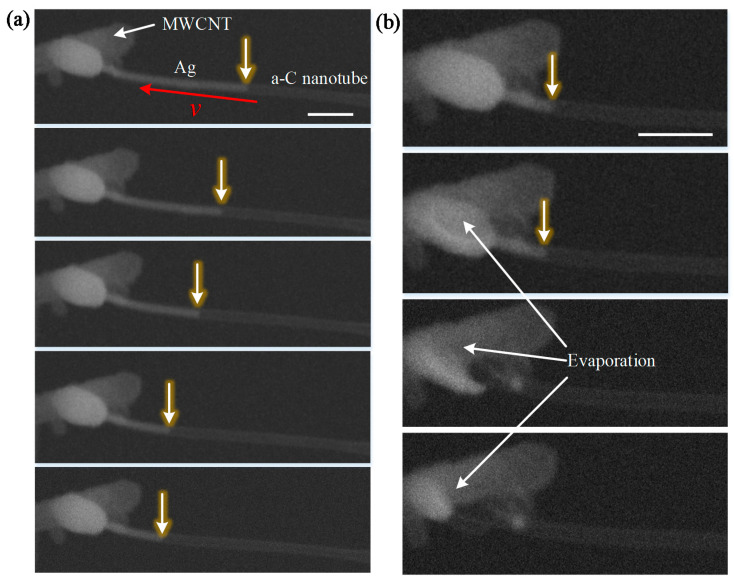
Reconfigurable nanointerconnection between MWCNT and Ag NW. (**a**) Nanointerconnection based on the encapsulated Ag in the a-C wrapped shell; (**b**). Evaporation disconnects the nanojunction and enables new interconnections. Scale bar: 300 nm.

Once we achieved the controllable melting and flow of Ag, we conducted a spot welding experiment to show its capabilities in nanowire interconnection. Figure 4a depicts the results of the interconnection. The setup is almost the same as Figure 1d. The only difference is that the Ag NW was pulled off at the tip during the picking up process, which leaves the terminal of the Ag NW open. By loading a proper bias voltage on this nanostructure, the encapsulated Ag will flow out controllably based on the electromigration force and thermal gradient force. Under these two forces, the flow of molten Ag inside the a-C shell can be noted by observing the white arrow marked in Figure 4a. Finally, an elliptical solder junction was formed at the tip of the a-C shell. As such, the MWCNT can be soldered together with the Ag NW. Moreover, the outflow Ag can also be removed by loading a higher bias voltage on this interconnected nanostructure to trigger evaporation, which enables error corrections (Figure 4b). The evaporation first occurred at the part of the elliptical solder joint and then gradually spread to all of the junction. The whole process took about 60 s, which provides us enough time to controllably modify the size or shape of the interconnect junction. After successfully removing the effluent Ag, the MWCNT can be reused in other applications.

## 4. Discussion

We can see that the proposed metal filling method in our research not only bypassed the complicated metal filling process but also acquired a highly efficient filling rate (~100%) compared to the already published methods [30,31]. The PVP-coated metallic Ag NW can be employed to the nanointerconnection directly without any complicated treatment. The conductive a-C shell was successfully formed spontaneously from the PVP layer before the melting and flow of the encapsulated Ag. Considering the high melting point of a-C (~3823 K, bulk material), nearly all the metal (Cu: ~1357 K; Au: ~1337 K; Pt: ~2041 K; Zn: ~692 K; W: 3695 K; Al: ~933 K, etc.) can be filled into the a-C shell and undergo the melting and flow treatment. The versatility of this proposed metal filling method is comparable to the developed welding method at macroscale, allowing the selection of interconnection materials based on the welding requirements. It is foreseeable that this metal-filled a-C shell can also be used in various promising applications such as nanoprinting [21], nanoscale circuit direct writing [32], and field effect transistor (FET) fabrication [33]. We believe that the proposed metal filling method, and its controllable melting and flow, open new possibilities for nanoscale interconnection. In the future, various metals should be filled into the a-C shell to meet the requirements of the materials in nanointerconnection. The a-C coated metallic NW can be automatically operated as a welding gun according to a geometric pattern that allows for outflow metal in both continuous and discrete modes as a function of the applied bias voltage.

## 5. Conclusions

In conclusion, using the in situ pyrolysis of the PVP-coated Ag NW, we have been able to obtain an Ag-filled a-C shell in which a controllable transfer of Ag from the inner part of the a-C shell onto an MWCNT can be performed. During this pyrolysis process, the observed Ag NW was 100% encapsulated into the self-formed a-C shell and completely flowed out when an appropriate voltage to the core–shell nanostructure was loaded. We also presented the formation mechanism of the Ag-filled a-C shell and implemented the characterization of the elements through EDS mapping. The controllable melting and flow of the encapsulated Ag have been also experimentally studied by injecting the Joule heat into the Ag NW via loading a proper DC bias voltage. The Ag flow velocity (rate) was found to be 38 nm/s (0.83 fg/s) at 30 µA and 928 nm/s (20.20 fg/s) at 50 µA. The subfemtogram mass transfer rate enables its ultrafine fabrication capabilities in nanofabrication. A reconfigurable nanointerconnection experiment was also implemented and the results demonstrated that the wrapped Ag could controllably flow out of the a-C shell when we broke the PVP layer on the tip during the Ag NW pick-up process. We therefore consider that this study is the proof-of-concept for the interconnection of material at the nanoscale based on the metal-filled a-C shell.
